# Systematic Evaluation of Four Cysteine Proteases (*Cs*CP1–4) from *Clonorchis sinensis* for Serodiagnosis: From Single-Antigen Screening to Multi-Antigen Modeling

**DOI:** 10.3390/tropicalmed11040103

**Published:** 2026-04-16

**Authors:** Shuai Wei, Xinyan Chen, Shangkun Cai, Xiaoqin Li, Ting Lu, Yaoting Li, Yuanlin Hou, Yanwen Li, Yunliang Shi

**Affiliations:** 1 Department of Clinical Laboratory, Hechi Hospital Affiliated to Youjiang Medical University for Nationalities (The People’s Hospital of Hechi), Key Laboratory of Foodborne Trematode Research and Prevention of Health Commission of Hechi, Hechi 547000, China; hcryws@163.com; 2 Modern Industrial College of Biomedicine and Great Health, Youjiang Medical University for Nationalities, Baise 533000, China; 3 Parasitology Department, School of Basic Medical Sciences, Guangxi Medical University, Nanning 530021, Chinalt2629558621@163.com (T.L.);; 4 Department of Clinical Laboratory, the First Affiliated Hospital of Guangxi Medical University, Key Laboratory of Clinical Laboratory Medicine of Guangxi Department of Education, Nanning 530021, China; cxy19970612@163.com; 5 Gastrointestinal Surgery, Hechi Hospital Affiliated to Youjiang Medical University for Nationalities, Hechi 547000, China; hcrycsk@163.com; 6 Key Laboratory of Basic Research on Regional Diseases (Guangxi Medical University), Education Department of Guangxi Zhuang Autonomous Region, Nanning 530021, China; 7 University Engineering Research Center of Advanced Technologies in Medical and Biological Intelligent Manufacturing of Guangxi, Nanning 530021, China

**Keywords:** *C. sinensis*, cysteine protease, diagnostic antigen, antibody subclass, indirect ELISA, serological diagnosis, combined detection

## Abstract

Background: Cysteine proteases of *Clonorchis sinensis* are potential diagnostic antigens, yet the performance of individual members within this diverse enzyme family requires systematic evaluation. This study aimed to assess the diagnostic potential of four recombinant cysteine proteases (r*Cs*CP1–4) for human clonorchiasis. Methods: An indirect ELISA was developed to measure serum reactivity (IgG, IgG subclasses, IgA) against r*Cs*CP1–4. The assay was validated using 180 microscopy-confirmed positive and 148 negative control sera. Samples were randomly split into training and validation sets (7.5:2.5). Diagnostic performance of single antigens and their combinations was evaluated using univariate and multivariate logistic regression and compared with a commercial kit. Key metrics included the area under the curve (AUC), sensitivity, specificity, accuracy, F1-score, and Kappa coefficient. Results: Four single antigen–antibody pairs showed high performance: r*Cs*CP1-IgG4 (AUC = 0.928), r*Cs*CP2-IgA (AUC = 0.863), r*Cs*CP3-IgG1 (AUC = 0.920), and r*Cs*CP4-IgG4 (AUC = 0.958). Among these, r*Cs*CP1-IgG4, r*Cs*CP3-IgG1, and r*Cs*CP4-IgG4 outperformed the commercial kit, achieving higher sensitivity (92.0%, 96.0%, 96.0% vs. 86.0%), specificity (87.5%, 81.3%, 90.6% vs. 78.1%), accuracy (92.0%, 88.9%, 94.1% vs. 86.0%), and F1-scores (0.902, 0.902, 0.939 vs. 0.829). The Kappa values for r*Cs*CP1-IgG4 (0.768) and r*Cs*CP4-IgG4 (0.773) indicated substantial agreement with the microscopic standard. Multi-antigen combinations (triple or quadruple) further enhanced performance, achieving sensitivity and specificity > 98% with an AUC approaching 1.0. Conclusions: This study identifies r*Cs*CP1 and r*Cs*CP4, particularly in combination with IgG4 detection, as highly promising diagnostic targets for clonorchiasis. Multi-antigen combinations significantly improved diagnostic performance compared to single-antigen assays, offering a strategy for high-precision diagnosis. Furthermore, the efficacy of the r*Cs*CP2-IgA pair suggests that detecting fecal secretory IgA could be a novel avenue for non-invasive, self-testing applications.

## 1. Introduction

*Clonorchis sinensis*, the Chinese liver fluke, is a foodborne parasitic trematode that infects the bile ducts of humans and other piscivorous mammals. Human infection occurs through the consumption of raw or undercooked freshwater fish harboring metacercariae. Chronic clonorchiasis is a major public health concern, associated with hepatobiliary pathologies such as cholelithiasis, cholangitis, and fibrosis, with severe cases progressing to cholangiocarcinoma—a malignancy for which *C. sinensis* has been classified as a Group I biological carcinogen by the International Agency for Research on Cancer since 2009 [1]. China bears the highest global disease burden of clonorchiasis. National epidemiological survey data from 2015 indicate a weighted *C. sinensis* infection rate of 0.47%, corresponding to an estimated 5.98 million infected individuals. The disease poses a significant public health challenge in endemic regions [2].

Accurate diagnosis is fundamental for disease management and control. Microscopic detection of eggs in stool remains the diagnostic gold standard; however, its sensitivity is limited in cases of light or early-stage infection, often leading to underdiagnosis [3]. Serological methods, particularly enzyme-linked immunosorbent assays (ELISAs), offer a more sensitive alternative. Nonetheless, the diagnostic performance of current serological tests remains suboptimal, largely constrained by the variable accuracy of available antigenic targets [4]. Therefore, the identification and validation of novel, highly immunoreactive antigens are critical for advancing the immunodiagnosis of clonorchiasis.

Cysteine proteases, key enzymes involved in parasite nutrition and development, have emerged as promising diagnostic candidates for several helminthic diseases. Studies on *Fasciola gigantica* [5], *F. hepatica* [6], *Spirometra mansoni* [7], and *Taenia solium* [8] have reported recombinant cysteine proteases with high diagnostic sensitivity and specificity. This collective evidence underscores the significant diagnostic potential of this enzyme family. However, this potential is not uniformly realized across all members of the cysteine protease family due to substantial sequence and functional diversity within the family. Notably, while diagnostic studies on *C. sinensis* cysteine proteases have been conducted, the reported sensitivity and specificity have been less satisfactory compared to those for other trematodes [9,10,11], indicating that the diagnostic utility of individual proteases requires careful, member-specific evaluation.

In our previous work, we successfully cloned and expressed four cysteine protease genes (*Cs*CP1–4) from *C. sinensis* and preliminarily characterized their immunogenicity [12]. Building on this foundation, this study presents the first systematic, head-to-head evaluation of the serodiagnostic potential of these four recombinant proteins (r*Cs*CP1–4). This is achieved by comprehensively profiling, for the first time, the specific antibody subclass responses (IgG, IgG1, IgG2a, IgG4, and IgA) against these antigens. The aim is to identify which specific antigen–antibody combinations exhibit superior diagnostic accuracy, thereby screening for high-value candidates suitable for developing improved serological diagnostic tools.

## 2. Materials and Methods

### 2.1. Recombinant Proteins

The four *C. sinensis* cysteine proteases involved in this study (IDs: ABJ89814.1, ABJ89818.1, ABK91809.1, and ABJ89815.1) were designated as *Cs*CP1, *Cs*CP2, *Cs*CP3, and *Cs*CP4 (collectively *Cs*CP1–4). In previous studies, we have completed the expression of the aforementioned recombinant proteins (r*Cs*CP1–4) [12,13] and stored them at −70 °C in the Department of Parasitology, Basic Medical College, Guangxi Medical University.

### 2.2. Human Serum Specimens

Positive sample: Sera obtained from patients with microscopy-confirmed *C. sinensis* eggs in their stool samples. Negative controls: Sera obtained from healthy individuals who tested negative for *C. sinensis* eggs in three consecutive stool examinations.

Serum samples were collected from adult subjects who visited the Hechi Hospital Affiliated to Youjiang Medical University for Nationalities between January 2020 and December 2024. For sample collection, subjects were instructed to fast overnight and sit upright. Venous blood (5 mL) was drawn from the antecubital fossa using a non-anticoagulant vacuum tube, followed by gentle inversion (5 times) to mix; The blood was allowed to clot at room temperature for 30 min, serum was separated by centrifugation at 3000 rpm for 15 min; The upper pale yellow serum layer was transferred to pre-labeled cryovials and immediately stored in a −80 °C ultra-low temperature freezer pending testing. All procedures adhered strictly to aseptic techniques.

Exclusion criteria included pregnant, infected with HIV, had malignant tumors, immunodeficiency disorders, or autoimmune diseases, or were undergoing immunosuppressive/immunomodulatory therapy.

### 2.3. Indirect ELISA to Detect the Specific Antibody

Wells were coated with 5 µg (a concentration optimized based on prior checkerboard titration in our lab) of recombinant protein and incubated at 4 °C for 12 h. Blocking was performed with 5% skim milk solution at 37 °C in the dark for 2 h. Diluted patient serum (1:200) was added and incubated at 4 °C for 12 h. All reagents were added in triplicate for each plate; the intra-assay coefficients of variation (CVs) for the quality control samples were maintained below 10%. After washing five times with PBS-T (pH 7.4), the following horseradish peroxidase (HRP)-conjugated secondary antibodies were added at 1:25,000 dilution and incubated at 37 °C in the dark for 1 h: Rabbit Anti-Human IgG (H+L)-HRP (Southernbiotech, Birmingham, AL, USA, 6140-05), Mouse Anti-Human IgG1 Fc-HRP (HP6001) (Southernbiotech, Birmingham, AL, USA, 9054-05), Mouse Anti-Human IgG2a Fc-HRP (31-7-4) (Southernbiotech, Birmingham, AL, USA, 9060-05), Mouse Anti-Human IgG4 Fc-HRP (HP6025) (Southernbiotech, Birmingham, AL, USA, 9200-05), and Goat Anti-Human IgA-HRP (Southernbiotech, Birmingham, AL, USA, 2050-05). After another five washes with PBS-T, 3,3′,5,5′-tetramethylbenzidine (TMB) substrate solution was added for color development in the dark for 10–15 min, followed by termination with 1 M H_2_SO_4_ solution. Absorbance was measured at 450 nm. Detection groups were named based on the recombinant protein and the specific antibody detected, e.g., “r*Cs*CP1-IgG” for the group using r*Cs*CP1 to detect serum-specific IgG.

The commercial *C. sinensis*-specific IgG antibody detection kit (Shenzhen Huakang Biomedical Engineering Co., Ltd., Shenzhen, China) was used strictly according to the manufacturer’s instructions (detailed information regarding the specific antigen and antibody used in the commercial kit could not be obtained, likely due to commercial confidentiality).

### 2.4. Diagnostic Efficacy Evaluation

The dataset was randomly split into an independent training and a validation set, at a 7.5:2.5 ratio. In the training set, univariate logistic regression was performed for each detection group to generate receiver operating characteristic (ROC) curves. The optimal cut-off value was determined by maximizing Youden’s index. Detection groups with an area under the curve (AUC) > 0.8 were selected for further analysis. Multivariate logistic regression was then applied to evaluate the diagnostic performance of multi-antigen combinations. Diagnostic efficacy was assessed using the following metrics: AUC, sensitivity, specificity, accuracy, F1-score, and Cohen’s Kappa coefficient.

### 2.5. Statistical Analysis

Categorical variables are described as percentages (%), and normally distributed continuous variables as mean ± standard deviation (mean ± SD). Comparisons of categorical variables were performed using the chi-square test, Fisher’s exact test, or McNemar’s test, as appropriate. Normally distributed continuous variables were compared using the independent samples *t*-test. For multiple comparisons between groups, pairwise testing was performed with Bonferroni correction. The diagnostic performance was evaluated using logistic regression. For the performance evaluation of the combined detection, Multicollinearity among the predictor variables was assessed using the variance inflation factor (VIF). A VIF value of <5 was considered to indicate no substantial multicollinearity. Comparisons of AUCs were conducted using the DeLong test. All analyses were conducted using )SPSS (Version 26.0; IBM, Armonk, NY, USA). A two-tailed *p*-value < 0.05 was considered statistically significant.

### 2.6. Ethical Considerations

Ethical approval for this research was granted by the Hechi Hospital Affiliated to Youjiang Medical University for Nationalities (protocol number: KY2024-063-01; date of approval: 4 March 2024). A broad consent form was obtained from all participants prior to enrolment. All study procedures, including participant recruitment, sample collection, and data handling, adhered to strict national ethical guidelines provided by the Declaration of Helsinki.

## 3. Results

### 3.1. Patient Demographics and Baseline Characteristics

A total of 328 participants were included in the study: 180 positive and 148 negative individuals. The dataset was randomly divided into independent training and validation sets at a 7.5:2.5 ratio, consisting of 246 and 82 participants, respectively. The two sets were comparable in baseline characteristics. The mean (SD) age was 41 (12) years in both groups (*p* = 0.812). No statistically significant differences were observed in sex distribution (*p* = 0.286) or the gold standard result (*p* = 0.200) between the training and validation sets (Table 1). These results confirm that the two sets were well-balanced in terms of baseline features.

### 3.2. Recombinant Protein Diagnostic Efficacy Evaluation

**Figure 1:**
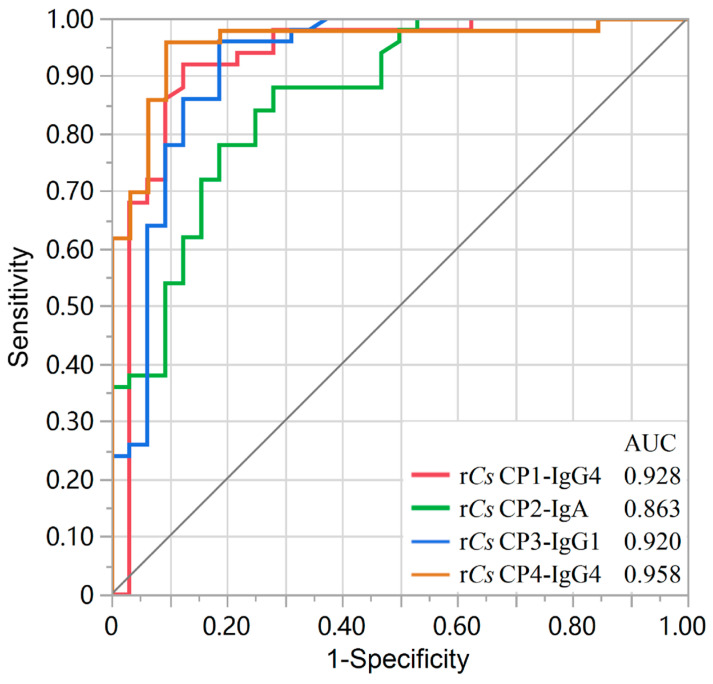
ROC curves for the four detection groups with high performance (AUC > 0.80).

Univariate logistic regression analysis was performed to evaluate the diagnostic performance of each recombinant protein against different antibody isotypes (Table 2). Four antigen–antibody pairs demonstrated high diagnostic accuracy, with an area under the curve (AUC) > 0.80: r*Cs*CP1-IgG4 (AUC = 0.928), r*Cs*CP2-IgA (AUC = 0.863), r*Cs*CP3-IgG1 (AUC = 0.920), and r*Cs*CP4-IgG4 (AUC = 0.958) (Figure 1). Pairwise comparisons of the AUCs among these four groups revealed that, although the difference did not reach statistical significance (*p* > 0.05), there was a trend suggesting a higher AUC for rCsCP4-IgG4. (Table 3).

### 3.3. Comparison with Commercial Kit

The validation set results indicated that, except for r*Cs*CP2-IgA, the other three groups showed higher numerical values in sensitivity, specificity, accuracy, and F1 score than the commercial kit. However, extended McNemar’s chi-square tests showed that these differences were not statistically significant (*p* > 0.05) with the current sample size (Table 4). Kappa consistency analysis showed that r*Cs*CP1-IgG4 (κ = 0.768) and r*Cs*CP4-IgG4 (κ = 0.773) had substantial agreement with the reference standard (0.61 < κ < 0.80), which was higher than that of the commercial kit (κ = 0.641) (Table 5).

### 3.4. Evaluation of Combined Detection Efficacy

Prior to the multifactorial logistic regression analysis, we assessed multicollinearity among the predictor variables. The variance inflation factor for all variables was less than 5, indicating no severe multicollinearity issues.

**Figure 2:**
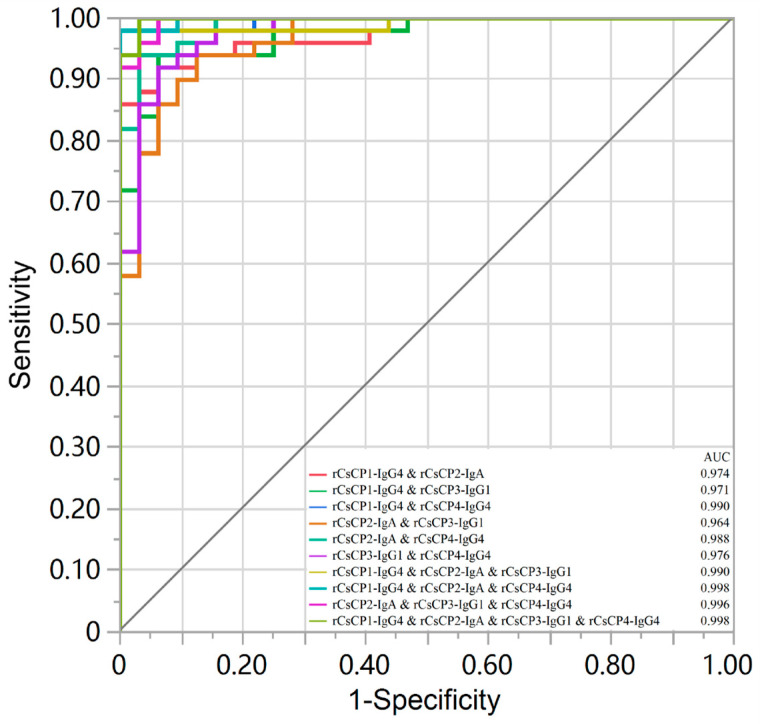
ROC curves for the combined detection.

Internal validation demonstrated that all combined detection strategies exhibited high diagnostic efficacy, with AUCs > 0.95 (Table 6, Figure 2). Sensitivity, specificity, accuracy, and F1 scores were superior to the commercial kit. Notably, the triple combination (r*Cs*CP1-IgG4 & r*Cs*CP2-IgA & r*Cs*CP4-IgG4) and the quadruple combination (r*Cs*CP1-IgG4 & r*Cs*CP2-IgA & r*Cs*CP3-IgG1 & r*Cs*CP4-IgG4) achieved remarkably high sensitivity, specificity, and accuracy values of 0.980, 1.000, and 0.988 and 1.000, 0.969, and 0.988, respectively, highlighting the advantage of a multi-antigen approach. These findings highlight the advantages of combined detection.

**Table 6:** Evaluation of the combined detection efficacy.

Combined Detection	AUC (95%CI)	Cutoff Value	TP	FN	FP	TN	Sensitivity (95%CI)	Specificity (95%CI)	Accuracy	F1 Score
r*Cs*CP1-IgG4 & r*Cs*CP2-IgA	0.974 (0.926–0.991)	0.592	47	3	4	28	0.940 (0.835–0.987)	0.875 (0.710–0.965)	0.915	0.931
r*Cs*CP1-IgG4 & r*Cs*CP3-IgG1	0.971 (0.920–0.990)	0.691	47	3	2	30	0.940 (0.835–0.987)	0.938 (0.792–0.992)	0.939	0.949
r*Cs*CP1-IgG4 & r*Cs*CP4-IgG4	0.990 (0.963–0.997)	0.373	49	1	5	27	0.980 (0.894–0.999)	0.844 (0.672–0.947)	0.927	0.942
r*Cs*CP2-IgA & r*Cs*CP3-IgG1	0.964 (0.907–0.987)	0.424	47	3	5	27	0.940 (0.835–0.987)	0.844 (0.672–0.947)	0.902	0.922
r*Cs*CP2-IgA & r*Cs*CP4-IgG4	0.988 (0.957–0.997)	0.532	47	3	1	31	0.940 (0.835–0.987)	0.969 (0.838–0.999)	0.951	0.959
r*Cs*CP3-IgG1 & r*Cs*CP4-IgG4	0.976 (0.925–0.993)	0.564	47	3	3	29	0.940 (0.835–0.987)	0.906 (0.750–0.980)	0.927	0.940
r*Cs*CP1-IgG4 & r*Cs*CP2-IgA & r*Cs*CP3-IgG1	0.990 (0.943–0.998)	0.462	49	1	1	31	0.980 (0.894–0.999)	0.969 (0.828–0.999)	0.976	0.980
r*Cs*CP1-IgG4 & r*Cs*CP2-IgA & r*Cs*CP4-IgG4	0.998 (0.983–1.000)	0.599	49	1	0	32	0.980 (0.894–0.999)	1.000 (0.891–1.000)	0.988	0.990
r*Cs*CP2-IgA & r*Cs*CP3-IgG1 & r*Cs*CP4-IgG4	0.996 (0.979–0.999)	0.449	49	1	2	30	0.980 (0.894–0.999)	0.938 (0.792–0.992)	0.963	0.970
r*Cs*CP1-IgG4 & r*Cs*CP2-IgA & r*Cs*CP3-IgG1 & r*Cs*CP4-IgG4	0.998 (0.982–1.000)	0.424	50	0	1	31	1.000 (0.929–1.000)	0.969 (0.838–0.999)	0.988	0.990

TP = True Positive; FN = False Negative; FP = False Positive; TN = True Negative.

## 4. Discussion

This study presents a systematic evaluation of the diagnostic potential of four cysteine proteases (*Cs*CP1–4) from *C. sinensis*. We identified distinct diagnostic profiles among these family members and their corresponding antibody isotypes. Notably, four specific antigen–antibody pairs—r*Cs*CP1-IgG4, r*Cs*CP2-IgA, r*Cs*CP3-IgG1, and r*Cs*CP4-IgG4—demonstrated superior performance (AUC > 0.85). Among these, r*Cs*CP1 and r*Cs*CP4 emerged as particularly promising candidates for IgG4 detection, achieving AUCs of 0.928 and 0.958, respectively.

The superior performance of r*Cs*CP1 and r*Cs*CP4 may be linked to their intrinsic structural properties. Previous studies suggest that compared to *Cs*CP2 and *Cs*CP3, *Cs*CP1 and *Cs*CP4 possess a greater abundance of both linear and conformational epitopes, along with a higher proportion of hydrophobic amino acids [14,15]. These features likely enhance their immunoreactivity and stability in antigen–antibody complexes, contributing to their higher diagnostic accuracy.

The high diagnostic potential of the IgG4 subclass is likely related to the immune response in chronic *C. sinensis* infection. *Cs*CP1–4 are expressed across various developmental stages and are widely distributed in the adult worm [12,13]. Under prolonged antigen stimulation, the immune system tends to undergo antibody class switching towards IgG4, a subtype associated with chronic inflammation. This could explain why IgG4 exhibits a higher sensitivity and specificity than other antibodies (e.g., IgG2a, IgA) in serodiagnosis.

Unlike previous studies focusing on single antigens or antibodies, our strategy of screening optimal “antigen–antibody” combinations more precisely identifies the dominant immune responses elicited by different antigens. In our study, multivariate logistic regression confirmed that combined detection can significantly enhance diagnostic performance; triple and quadruple combinations achieved excellent discrimination (AUC approaching 1.0), with sensitivity and specificity values exceeding 98%, outperforming the commercial kit. This indicates that a multi-antigen model can overcome the limitations of single antigens, representing a key direction for developing highly accurate serological diagnostics.

Although serum monomeric IgA generally shows weaker systemic immunoreactivity, the rCsCP2-IgA pair displays promising diagnostic potential (AUC = 0.863). This result prompts consideration of alternative diagnostic compartments. Secretory IgA (sIgA) is a cornerstone of mucosal immunity, and *C. sinensis* infection is known to upregulate sIgA expression in the biliary epithelium [16]. We therefore hypothesize that detecting *Cs*CP2-specific sIgA in fecal samples might hold greater diagnostic value than serum IgA. Given the stability of sIgA in the gut environment and existing standardized detection protocols [17,18,19], these findings warrant future investigation into the potential role of fecal sIgA as a non-invasive diagnostic marker. Such an innovation could decentralize testing and reduce reliance on clinical infrastructure [20].

## 5. Limitations

Several limitations of this study warrant discussion. Firstly, the imperfect sensitivity of the microscopic gold standard (fecal examination) means that some negative controls might harbor light, undetected infections. This could lead to an underestimation of our assay’s specificity. Furthermore, the positive results of this gold standard rely solely on the examiner’s subjective judgment and have not been confirmed by PCR; this may lead to the misidentification of other parasite eggs as *Clonorchis sinensis*. Consequently, the overall diagnostic sensitivity of our assay may be underestimated. Secondly, the lack of testing with sera from patients infected with other parasites (such as paragonimiasis, fascioliasis, and schistosomiasis) or from individuals with autoimmune diseases restricts a full assessment of the diagnostic specificity of these recombinant antigens. Future work should systematically incorporate such serum panels to evaluate potential cross-reactivity of the identified optimal antigen–antibody combinations with antibodies against other common parasites, which is essential for establishing robust assay specificity. Thirdly, the microscopy-confirmed positive samples were not stratified by infection intensity. Consequently, we could not evaluate the correlation between the magnitude of the serological response and the level of infection. Assessing this relationship is important for understanding the potential utility of these markers in monitoring treatment efficacy or reflecting disease burden. Fourthly, validation with larger cohorts, particularly including patients with early-stage or low-intensity infections, is needed to confirm diagnostic sensitivity across the disease spectrum. Fifthly, all serum samples in this study were collected from a single center and represent a single, cross-sectional measurement without longitudinal follow-up. Future studies should incorporate samples from more diverse geographical sources and conduct longitudinal investigations to validate the generalizability of these diagnostic markers and their dynamic changes throughout the infection process. Finally, due to sample volume constraints, the synergistic effects of the multi-antigen combinations were evaluated statistically but not confirmed through experimental additive or blocking assays.

## 6. Conclusions

Our findings identify r*Cs*CP1 and r*Cs*CP4 as highly promising diagnostic antigens for detecting IgG4 antibodies against *C. sinensis*. We further show that a multi-antigen combination substantially improves diagnostic accuracy. Beyond serum-based diagnostics, our findings regarding the rCsCP2-IgA pair suggest the potential for developing non-invasive tests. Future work should explore fecal sIgA as a promising diagnostic marker.

## Figures and Tables

**Table 1:** Patient demographics and baseline characteristics.

Characteristic	Training Set *n* = 246	Validation Set *n* = 82	*p*
Sex, *n* (%)			0.286 ^1^
Male	174 (70.73)	63 (76.83)	
Female	72 (29.27)	19 (23.17)	
Age, Mean ± SD	41 ± 12	41 ± 12	0.812 ^2^
Gold standard result, *n* (%)			0.200 ^1^
Positive	130 (52.85)	50 (60.98)	
Negative	116 (47.15)	32 (39.02)	

^1^ Pearson’s Chi-squared test; ^2^ Wilcoxon rank sum test.

**Table 2:** The AUC of serum-specific antibodies detected by recombinant proteins.

Recombinant Protein	Specific Antibody				
	IgG	IgG1	IgG2a	IgG4	IgA
r*Cs*CP1	0.732	0.781	0.554	0.928	0.667
r*Cs*CP2	0.565	0.693	0.582	0.776	0.863
r*Cs*CP3	0.531	0.920	0.544	0.787	0.591
r*Cs*CP4	0.702	0.767	0.508	0.958	0.633

**Table 3:** Comparison of AUCs using the DeLong test.

Detection Group		AUC Difference	χ^2^	*p*
r*Cs*CP1-IgG4	r*Cs*CP2-IgA	0.065	1.332	0.248
r*Cs*CP1-IgG4	r*Cs*CP3-IgG1	0.008	0.021	0.884
r*Cs*CP1-IgG4	r*Cs*CP4-IgG4	−0.030	0.477	0.490
r*Cs*CP2-IgA	r*Cs*CP3-IgG1	−0.058	0.981	0.322
r*Cs*CP2-IgA	r*Cs*CP4-IgG4	−0.095	3.657	0.056
r*Cs*CP3-IgG1	r*Cs*CP4-IgG4	−0.037	0.808	0.369

**Table 4:** Evaluation of the detection efficacy of high-performance detection groups and a comparison with the commercial kit.

Detection Group	AUC (95%CI)	Cutoff Value	TP	FN	FP	TN	Sensitivity (95%CI)	Specificity (95%CI)	Accuracy	F1 Score	Sensitivity		Specificity	
											χ^2^	*p*	*χ^2^*	*p*
Commercial Kit	-	-	43	7	7	25	0.860 (0.733–0.942)	0.781 (0.600–0.907)	0.860	0.829	-	-	-	-
r*Cs*CP1-IgG4	0.928 (0.819–0.973)	0.66	46	4	4	28	0.920 (0.808–0.978)	0.875 (0.710–0.965)	0.920	0.902	0.818	0.366	0.500	0.480
r*Cs*CP2-IgA	0.863 (0.760–0.926)	0.84	42	8	8	24	0.840 (0.709–0.928)	0.750 (0.566–0.885)	0.840	0.805	0.077	0.782	0.400	0.527
r*Cs*CP3-IgG1	0.920 (0.817–0.968)	0.25	48	2	6	26	0.960 (0.863–0.995)	0.813 (0.636–0.928)	0.889	0.902	1.000	0.317	1.000	0.317
r*Cs*CP4-IgG4	0.958 (0.884–0.985)	0.93	48	2	3	29	0.960 (0.863–0.995)	0.906 (0.750–0.980)	0.941	0.939	0.400	0.527	2.667	0.103

TP = True Positive; FN = False Negative; FP = False Positive; TN = True Negative.

**Table 5:** Inter-rater reliability between high-performance detection groups and the commercial kit for the gold standard using Cohen’s Kappa.

Detection Group	κ (95%CI)	*p*
Commercial Kit	0.641 (0.471–0.812)	<0.001
r*Cs*CP1-IgG4	0.768 (0.625–0.911)	<0.001
r*Cs*CP2-IgA	0.567 (0.384–0.746)	<0.001
r*Cs*CP3-IgG1	0.630 (0.464–0.797)	<0.001
r*Cs*CP4-IgG4	0.773 (0.634–0.912)	<0.001

## Data Availability

The data presented in this study are available on request from the corresponding author. The data are not publicly available due to privacy and ethical restrictions.
